# Diaqua­(1,4,8,11-tetra­aza­cyclo­tetra­decane-κ^4^ *N* ^1^,*N* ^4^,*N* ^8^,*N* ^11^)copper(II) dideca­noate dihydrate

**DOI:** 10.1107/S1600536810025699

**Published:** 2010-07-07

**Authors:** Nur Syamimi Ahmad Tajidi, Norbani Abdullah, Zainudin Arifin, Kong Wai Tan, Seik Weng Ng

**Affiliations:** a Department of Chemistry, University of Malaya, 50603 Kuala Lumpur, Malaysia

## Abstract

The Cu^II^ atom in the title salt, [Cu(C_10_H_24_N_4_)(H_2_O)_2_][CH_3_(CH_2_)_8_CO_2_]_2_·2H_2_O, is chelated by the four N atoms of the 1,4,8,11-tetra­aza­cyclo­tetra­decane (cyclam) ligand and is coordinated by two water mol­ecules in a Jahn–Teller-type tetra­gonally distorted octa­hedral geometry. The Cu^II^ atom lies on a center of inversion. The cations, anions and uncoordinated water mol­ecules are linked by N—H⋯O and O—H⋯O hydrogen bonds, forming a layer structure parallel to (001).

## Related literature

For related (1,4,8,11-tetra­aza­cyclo­tetra­deca­ne)copper carb­oxy­l­ates, see: Lindoy *et al.* (2003 ▶); Hunter *et al.* (2005 ▶). 
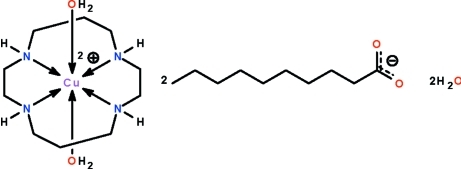


## Experimental

### 

#### Crystal data

[Cu(C_10_H_24_N_4_)(H_2_O)_2_](C_10_H_19_O_2_)_2_·2H_2_O
*M* *_r_* = 678.44
Triclinic, 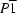

*a* = 6.9820 (6) Å
*b* = 8.8006 (8) Å
*c* = 15.3291 (13) Å
α = 95.045 (1)°
β = 93.158 (1)°
γ = 98.423 (1)°
*V* = 925.93 (14) Å^3^
*Z* = 1
Mo *K*α radiation
μ = 0.64 mm^−1^
*T* = 100 K
0.30 × 0.20 × 0.02 mm

#### Data collection

Bruker SMART APEX diffractometer
Absorption correction: multi-scan (*SADABS*; Sheldrick, 1996 ▶) *T*_min_ = 0.832, *T*_max_ = 0.987
8967 measured reflections
4230 independent reflections
3736 reflections with *I* > 2σ(*I*)
*R*_int_ = 0.034

#### Refinement

*R*[*F* ^2^ > 2σ(*F* ^2^)] = 0.035
*wR*(*F* ^2^) = 0.086
*S* = 1.06
4230 reflections
220 parameters
6 restraints
H atoms treated by a mixture of independent and constrained refinement
Δρ_max_ = 0.33 e Å^−3^
Δρ_min_ = −0.43 e Å^−3^

### 

Data collection: *APEX2* (Bruker, 2009 ▶); cell refinement: *SAINT* (Bruker, 2009 ▶); data reduction: *SAINT*; program(s) used to solve structure: *SHELXS97* (Sheldrick, 2008 ▶); program(s) used to refine structure: *SHELXL97* (Sheldrick, 2008 ▶); molecular graphics: *X-SEED* (Barbour, 2001 ▶); software used to prepare material for publication: * publCIF*  (Westrip, 2010 ▶).

## Supplementary Material

Crystal structure: contains datablocks global, I. DOI: 10.1107/S1600536810025699/bt5286sup1.cif

Structure factors: contains datablocks I. DOI: 10.1107/S1600536810025699/bt5286Isup2.hkl

Additional supplementary materials:  crystallographic information; 3D view; checkCIF report

## Figures and Tables

**Table 1:** Selected bond lengths (Å)

Cu1—N1	2.029 (1)
Cu1—N2	2.000 (1)
Cu1—O1w	2.443 (1)

**Table 2:** Hydrogen-bond geometry (Å, °)

*D*—H⋯*A*	*D*—H	H⋯*A*	*D*⋯*A*	*D*—H⋯*A*
N1—H1⋯O2	0.86 (1)	2.30 (1)	3.025 (2)	144 (2)
N2—H2⋯O2w^i^	0.85 (1)	2.18 (1)	2.974 (2)	154 (2)
O1w—H11⋯O2^i^	0.83 (1)	1.95 (1)	2.774 (2)	172 (2)
O1w—H12⋯O2w	0.83 (1)	1.98 (1)	2.799 (2)	169 (2)
O2w—H21⋯O1	0.83 (1)	1.86 (1)	2.694 (2)	177 (2)
O2w—H22⋯O1^ii^	0.83 (1)	1.97 (1)	2.771 (2)	163 (2)

Symmetry codes: (i) 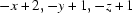; (ii) 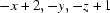.

**Fig. 1.**
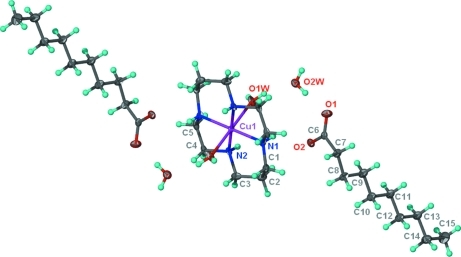
Anisotropic displacement ellipsoid plot (Barbour, 2001) of [Cu(H2O)2(C10H24N4)]2+ 2[CH3(CH2)8CO2]-.2H2O at the 70% probability level; hydrogen atoms are drawn as spheres of arbitrary radius.

## supplementary crystallographic information

### Comment

The copper(II) ion forms a number of complexes with 1,4,8,11-tetraazacyclotetradecane in which the metal atom is coordinated by the four amino donor-atoms of the cyclic ligand. Among the carboxylate derivatives, neither the acetate nor the benzoate ions bind directly with the copper atom. The copper atom is coordinated instead by water molecules so that the carboxylate group interacts indirectly with the metal atom through the coordinated water molecules (Hunter *et al.*, 2005; Lindoy *et al.*, 2003). The copper(II) atom in the salt, [Cu(H_2_O)_2_(C_10_H_24_N_4_)]^2+^ 2[CH_3_(CH_2_)_8_CO_2_]^-.^2H_2_O (Scheme I), is chelated by the four nitrogen atoms of the cyclam ligand and is coordinated by two water molecules in a Jahn-Teller type of tetragonally distorted octahedral geometry. The copper atom lies on a center of inversion (Fig. 1). The cations, anions and lattice water molecules are linked by N–H···O and O–H···O hydrogen bonds to form a layer structure.

### Experimental

1,4,8,11-Tetraazacyclotetradecane (0.50 g, 2.50 mmol) dissolved in ethanol (25 ml) was mixed with a suspension of copper decanoate (1.01.80 g, 2.5 mmol) in ethanol (50 ml) to give a purple solution. The solution was heated for an hour and then filtered. Prismatic crystals separated from the solution when it was left to cool slowly.

### Refinement

Carbon-bound H-atoms were placed in calculated positions (C—H 0.95 to 0.98 Å) and were included in the refinement in the riding model approximation, with *U*(H) set to 1.2 to 1.5*U*(C).

The amino and water H-atoms were located in a difference Fourier map, and were refined with distance restraints of N–H 0.86±0.01, O–H 0.84±0.01 Å; their displacement parameters were freely refined.

### Crystal data

**Table:**

[Cu(C_10_H_24_N_4_)(H_2_O)_2_](C_10_H_19_O_2_)_2_·2H_2_O	*Z* = 1
*M**_r_* = 678.44	*F*(000) = 371
Triclinic, *P*1	*D*_x_ = 1.217 Mg m^−^^3^
Hall symbol: -P 1	Mo *K*α radiation, λ = 0.71073 Å
*a* = 6.9820 (6) Å	Cell parameters from 3079 reflections
*b* = 8.8006 (8) Å	θ = 2.4–28.1°
*c* = 15.3291 (13) Å	µ = 0.64 mm^−^^1^
α = 95.045 (1)°	*T* = 100 K
β = 93.158 (1)°	Plate, purple
γ = 98.423 (1)°	0.30 × 0.20 × 0.02 mm
*V* = 925.93 (14) Å^3^	

### Data collection

**Table:**

Bruker SMART APEX diffractometer	4230 independent reflections
Radiation source: fine-focus sealed tube	3736 reflections with *I* > 2σ(*I*)
graphite	*R*_int_ = 0.034
ω scans	θ_max_ = 27.5°, θ_min_ = 1.3°
Absorption correction: multi-scan (*SADABS*; Sheldrick, 1996)	*h* = −9→9
*T*_min_ = 0.832, *T*_max_ = 0.987	*k* = −11→11
8967 measured reflections	*l* = −19→18

### Refinement

**Table:**

Refinement on *F*^2^	Primary atom site location: structure-invariant direct methods
Least-squares matrix: full	Secondary atom site location: difference Fourier map
*R*[*F*^2^ > 2σ(*F*^2^)] = 0.035	Hydrogen site location: inferred from neighbouring sites
*wR*(*F*^2^) = 0.086	H atoms treated by a mixture of independent and constrained refinement
*S* = 1.06	*w* = 1/[σ^2^(*F*_o_^2^) + (0.0348*P*)^2^ + 0.1911*P*] where *P* = (*F*_o_^2^ + 2*F*_c_^2^)/3
4230 reflections	(Δ/σ)_max_ = 0.001
220 parameters	Δρ_max_ = 0.33 e Å^−^^3^
6 restraints	Δρ_min_ = −0.43 e Å^−^^3^

### Fractional atomic coordinates and isotropic or equivalent isotropic displacement parameters (Å^2^)

**Table:**

	*x*	*y*	*z*	*U*_iso_*/*U*_eq_	
Cu1	0.5000	0.5000	0.5000	0.01105 (9)	
O1	0.9257 (2)	0.05590 (14)	0.38643 (8)	0.0242 (3)	
O2	0.93155 (17)	0.28063 (13)	0.33012 (8)	0.0179 (3)	
O1W	0.81745 (18)	0.48550 (14)	0.57137 (9)	0.0204 (3)	
H11	0.900 (3)	0.555 (2)	0.5970 (13)	0.035 (6)*	
H12	0.870 (3)	0.4065 (17)	0.5704 (15)	0.037 (7)*	
O2W	0.95494 (18)	0.20230 (14)	0.54959 (9)	0.0185 (3)	
H21	0.945 (3)	0.160 (2)	0.4983 (8)	0.040 (7)*	
H22	0.982 (3)	0.1320 (19)	0.5783 (13)	0.029 (6)*	
N1	0.55275 (19)	0.35212 (15)	0.39806 (9)	0.0135 (3)	
H1	0.6723 (15)	0.342 (2)	0.4045 (12)	0.022 (5)*	
N2	0.6209 (2)	0.68731 (15)	0.44674 (9)	0.0130 (3)	
H2	0.7437 (14)	0.692 (2)	0.4559 (12)	0.017 (5)*	
C1	0.5260 (3)	0.4008 (2)	0.30884 (11)	0.0184 (4)	
H1A	0.3879	0.4104	0.2963	0.022*	
H1B	0.5602	0.3211	0.2653	0.022*	
C2	0.6516 (3)	0.5543 (2)	0.29990 (11)	0.0200 (4)	
H2A	0.6509	0.5722	0.2370	0.024*	
H2B	0.7869	0.5480	0.3205	0.024*	
C3	0.5859 (3)	0.6912 (2)	0.35105 (11)	0.0186 (4)	
H3A	0.6570	0.7881	0.3332	0.022*	
H3B	0.4457	0.6902	0.3367	0.022*	
C4	0.5625 (2)	0.82300 (18)	0.49625 (12)	0.0179 (4)	
H4A	0.4297	0.8361	0.4754	0.021*	
H4B	0.6522	0.9174	0.4871	0.021*	
C5	0.5685 (2)	0.79793 (18)	0.59202 (12)	0.0181 (4)	
H5A	0.7039	0.7967	0.6145	0.022*	
H5B	0.5178	0.8826	0.6259	0.022*	
C6	0.9049 (2)	0.13621 (18)	0.32350 (11)	0.0137 (3)	
C7	0.8332 (3)	0.04950 (19)	0.23452 (11)	0.0172 (4)	
H7A	0.6895	0.0311	0.2309	0.021*	
H7B	0.8776	−0.0525	0.2313	0.021*	
C8	0.8998 (2)	0.1307 (2)	0.15509 (11)	0.0177 (4)	
H8A	0.8265	0.0760	0.1015	0.021*	
H8B	0.8690	0.2372	0.1613	0.021*	
C9	1.1168 (2)	0.1371 (2)	0.14399 (12)	0.0194 (4)	
H9A	1.1902	0.2006	0.1949	0.023*	
H9B	1.1499	0.0314	0.1437	0.023*	
C10	1.1795 (2)	0.2040 (2)	0.06012 (11)	0.0189 (4)	
H10A	1.1426	0.3084	0.0598	0.023*	
H10B	1.1078	0.1389	0.0093	0.023*	
C11	1.3965 (3)	0.2155 (2)	0.04841 (12)	0.0204 (4)	
H11A	1.4684	0.2866	0.0968	0.024*	
H11B	1.4356	0.1125	0.0525	0.024*	
C12	1.4536 (2)	0.2727 (2)	−0.03877 (11)	0.0188 (4)	
H12A	1.4084	0.3732	−0.0439	0.023*	
H12B	1.3857	0.1989	−0.0870	0.023*	
C13	1.6711 (3)	0.2921 (2)	−0.05047 (11)	0.0196 (4)	
H13A	1.7394	0.3678	−0.0032	0.024*	
H13B	1.7174	0.1922	−0.0444	0.024*	
C14	1.7228 (3)	0.3460 (2)	−0.13872 (12)	0.0253 (4)	
H14A	1.6723	0.4440	−0.1454	0.030*	
H14B	1.6570	0.2685	−0.1857	0.030*	
C15	1.9396 (3)	0.3709 (3)	−0.15119 (14)	0.0329 (5)	
H15A	1.9617	0.4047	−0.2095	0.049*	
H15B	1.9908	0.2740	−0.1458	0.049*	
H15C	2.0058	0.4501	−0.1062	0.049*	

### Atomic displacement parameters (Å^2^)

**Table:**

	*U*^11^	*U*^22^	*U*^33^	*U*^12^	*U*^13^	*U*^23^
Cu1	0.01256 (15)	0.00841 (14)	0.01241 (16)	0.00162 (10)	0.00342 (11)	0.00065 (10)
O1	0.0374 (8)	0.0185 (6)	0.0167 (7)	0.0035 (5)	−0.0007 (6)	0.0043 (5)
O2	0.0220 (6)	0.0131 (5)	0.0184 (6)	0.0051 (5)	−0.0005 (5)	−0.0012 (5)
O1W	0.0139 (6)	0.0158 (6)	0.0306 (8)	0.0045 (5)	−0.0032 (5)	−0.0027 (5)
O2W	0.0200 (6)	0.0159 (6)	0.0200 (7)	0.0046 (5)	0.0001 (5)	0.0010 (5)
N1	0.0089 (7)	0.0154 (7)	0.0157 (7)	0.0023 (5)	0.0020 (6)	−0.0020 (5)
N2	0.0096 (7)	0.0127 (6)	0.0176 (7)	0.0030 (5)	0.0031 (6)	0.0028 (5)
C1	0.0170 (9)	0.0250 (9)	0.0133 (9)	0.0061 (7)	0.0014 (7)	−0.0026 (7)
C2	0.0177 (9)	0.0310 (10)	0.0133 (9)	0.0062 (7)	0.0045 (7)	0.0074 (7)
C3	0.0170 (9)	0.0211 (8)	0.0193 (9)	0.0037 (7)	0.0017 (7)	0.0098 (7)
C4	0.0144 (8)	0.0085 (7)	0.0312 (10)	0.0019 (6)	0.0050 (7)	0.0014 (7)
C5	0.0148 (8)	0.0109 (7)	0.0270 (10)	0.0001 (6)	0.0039 (7)	−0.0052 (7)
C6	0.0098 (8)	0.0163 (8)	0.0155 (9)	0.0034 (6)	0.0040 (6)	−0.0002 (6)
C7	0.0184 (9)	0.0157 (8)	0.0163 (9)	−0.0006 (6)	0.0040 (7)	−0.0016 (7)
C8	0.0179 (9)	0.0211 (8)	0.0133 (8)	0.0003 (7)	0.0030 (7)	−0.0002 (7)
C9	0.0176 (9)	0.0240 (9)	0.0168 (9)	0.0027 (7)	0.0032 (7)	0.0017 (7)
C10	0.0174 (9)	0.0234 (9)	0.0156 (9)	0.0009 (7)	0.0036 (7)	0.0024 (7)
C11	0.0192 (9)	0.0259 (9)	0.0162 (9)	0.0025 (7)	0.0054 (7)	0.0021 (7)
C12	0.0165 (9)	0.0242 (9)	0.0150 (9)	0.0003 (7)	0.0031 (7)	0.0018 (7)
C13	0.0186 (9)	0.0250 (9)	0.0154 (9)	0.0024 (7)	0.0033 (7)	0.0027 (7)
C14	0.0210 (10)	0.0367 (11)	0.0182 (10)	0.0012 (8)	0.0044 (7)	0.0071 (8)
C15	0.0250 (10)	0.0443 (12)	0.0316 (12)	0.0039 (9)	0.0138 (9)	0.0104 (9)

### Geometric parameters (Å, °)

**Table:**

Cu1—N1	2.029 (1)	C5—H5B	0.9900
Cu1—N1^i^	2.029 (1)	C6—C7	1.524 (2)
Cu1—N2	2.000 (1)	C7—C8	1.526 (2)
Cu1—N2^i^	2.000 (1)	C7—H7A	0.9900
Cu1—O1w	2.443 (1)	C7—H7B	0.9900
O1—C6	1.259 (2)	C8—C9	1.527 (2)
O2—C6	1.2515 (19)	C8—H8A	0.9900
O1W—H11	0.832 (10)	C8—H8B	0.9900
O1W—H12	0.831 (9)	C9—C10	1.520 (2)
O2W—H21	0.834 (9)	C9—H9A	0.9900
O2W—H22	0.828 (9)	C9—H9B	0.9900
N1—C1	1.479 (2)	C10—C11	1.525 (2)
N1—C5^i^	1.485 (2)	C10—H10A	0.9900
N1—H1	0.855 (9)	C10—H10B	0.9900
N2—C3	1.478 (2)	C11—C12	1.522 (2)
N2—C4	1.478 (2)	C11—H11A	0.9900
N2—H2	0.854 (9)	C11—H11B	0.9900
C1—C2	1.520 (2)	C12—C13	1.525 (2)
C1—H1A	0.9900	C12—H12A	0.9900
C1—H1B	0.9900	C12—H12B	0.9900
C2—C3	1.521 (2)	C13—C14	1.517 (2)
C2—H2A	0.9900	C13—H13A	0.9900
C2—H2B	0.9900	C13—H13B	0.9900
C3—H3A	0.9900	C14—C15	1.522 (3)
C3—H3B	0.9900	C14—H14A	0.9900
C4—C5	1.503 (3)	C14—H14B	0.9900
C4—H4A	0.9900	C15—H15A	0.9800
C4—H4B	0.9900	C15—H15B	0.9800
C5—N1^i^	1.485 (2)	C15—H15C	0.9800
C5—H5A	0.9900		
			
N2—Cu1—N2^i^	180.000 (1)	O2—C6—C7	118.55 (15)
N2—Cu1—N1	93.73 (6)	O1—C6—C7	116.87 (14)
N2^i^—Cu1—N1	86.27 (6)	C6—C7—C8	115.27 (13)
N2—Cu1—N1^i^	86.27 (6)	C6—C7—H7A	108.5
N2^i^—Cu1—N1^i^	93.73 (6)	C8—C7—H7A	108.5
N1—Cu1—N1^i^	180.00 (5)	C6—C7—H7B	108.5
N2—Cu1—O1W	88.48 (5)	C8—C7—H7B	108.5
N2^i^—Cu1—O1W	91.52 (5)	H7A—C7—H7B	107.5
N1—Cu1—O1W	90.25 (5)	C9—C8—C7	113.07 (15)
N1^i^—Cu1—O1W	89.75 (5)	C9—C8—H8A	109.0
Cu1—O1W—H11	130.2 (16)	C7—C8—H8A	109.0
Cu1—O1W—H12	124.9 (16)	C9—C8—H8B	109.0
H11—O1W—H12	105 (2)	C7—C8—H8B	109.0
H21—O2W—H22	102 (2)	H8A—C8—H8B	107.8
C1—N1—C5^i^	112.07 (13)	C10—C9—C8	113.02 (15)
C1—N1—Cu1	117.07 (10)	C10—C9—H9A	109.0
C5^i^—N1—Cu1	106.17 (10)	C8—C9—H9A	109.0
C1—N1—H1	105.4 (13)	C10—C9—H9B	109.0
C5^i^—N1—H1	108.8 (13)	C8—C9—H9B	109.0
Cu1—N1—H1	107.0 (13)	H9A—C9—H9B	107.8
C3—N2—C4	111.44 (13)	C9—C10—C11	114.15 (15)
C3—N2—Cu1	117.71 (10)	C9—C10—H10A	108.7
C4—N2—Cu1	107.26 (10)	C11—C10—H10A	108.7
C3—N2—H2	105.7 (13)	C9—C10—H10B	108.7
C4—N2—H2	107.8 (13)	C11—C10—H10B	108.7
Cu1—N2—H2	106.5 (13)	H10A—C10—H10B	107.6
N1—C1—C2	111.30 (14)	C12—C11—C10	113.15 (15)
N1—C1—H1A	109.4	C12—C11—H11A	108.9
C2—C1—H1A	109.4	C10—C11—H11A	108.9
N1—C1—H1B	109.4	C12—C11—H11B	108.9
C2—C1—H1B	109.4	C10—C11—H11B	108.9
H1A—C1—H1B	108.0	H11A—C11—H11B	107.8
C1—C2—C3	113.84 (14)	C11—C12—C13	114.24 (15)
C1—C2—H2A	108.8	C11—C12—H12A	108.7
C3—C2—H2A	108.8	C13—C12—H12A	108.7
C1—C2—H2B	108.8	C11—C12—H12B	108.7
C3—C2—H2B	108.8	C13—C12—H12B	108.7
H2A—C2—H2B	107.7	H12A—C12—H12B	107.6
N2—C3—C2	111.53 (13)	C14—C13—C12	112.92 (15)
N2—C3—H3A	109.3	C14—C13—H13A	109.0
C2—C3—H3A	109.3	C12—C13—H13A	109.0
N2—C3—H3B	109.3	C14—C13—H13B	109.0
C2—C3—H3B	109.3	C12—C13—H13B	109.0
H3A—C3—H3B	108.0	H13A—C13—H13B	107.8
N2—C4—C5	108.50 (13)	C13—C14—C15	114.09 (16)
N2—C4—H4A	110.0	C13—C14—H14A	108.7
C5—C4—H4A	110.0	C15—C14—H14A	108.7
N2—C4—H4B	110.0	C13—C14—H14B	108.7
C5—C4—H4B	110.0	C15—C14—H14B	108.7
H4A—C4—H4B	108.4	H14A—C14—H14B	107.6
N1^i^—C5—C4	108.31 (13)	C14—C15—H15A	109.5
N1^i^—C5—H5A	110.0	C14—C15—H15B	109.5
C4—C5—H5A	110.0	H15A—C15—H15B	109.5
N1^i^—C5—H5B	110.0	C14—C15—H15C	109.5
C4—C5—H5B	110.0	H15A—C15—H15C	109.5
H5A—C5—H5B	108.4	H15B—C15—H15C	109.5
O2—C6—O1	124.52 (15)		
			
N2—Cu1—N1—C1	−39.47 (12)	C4—N2—C3—C2	178.27 (13)
N2^i^—Cu1—N1—C1	140.53 (12)	Cu1—N2—C3—C2	−57.23 (16)
O1W—Cu1—N1—C1	−127.96 (11)	C1—C2—C3—N2	70.00 (18)
N2—Cu1—N1—C5^i^	−165.46 (11)	C3—N2—C4—C5	170.11 (13)
N2^i^—Cu1—N1—C5^i^	14.54 (11)	Cu1—N2—C4—C5	39.93 (15)
O1W—Cu1—N1—C5^i^	106.05 (11)	N2—C4—C5—N1^i^	−54.24 (17)
N1—Cu1—N2—C3	39.47 (12)	O2—C6—C7—C8	31.5 (2)
N1^i^—Cu1—N2—C3	−140.53 (12)	O1—C6—C7—C8	−151.30 (16)
O1W—Cu1—N2—C3	129.61 (11)	C6—C7—C8—C9	69.42 (19)
N1—Cu1—N2—C4	166.02 (11)	C7—C8—C9—C10	174.47 (14)
N1^i^—Cu1—N2—C4	−13.98 (11)	C8—C9—C10—C11	178.58 (14)
O1W—Cu1—N2—C4	−103.84 (10)	C9—C10—C11—C12	176.14 (14)
C5^i^—N1—C1—C2	−179.84 (13)	C10—C11—C12—C13	177.40 (14)
Cu1—N1—C1—C2	57.16 (16)	C11—C12—C13—C14	178.81 (15)
N1—C1—C2—C3	−70.19 (19)	C12—C13—C14—C15	178.29 (16)

Symmetry codes: (i) −*x*+1, −*y*+1, −*z*+1.

### Hydrogen-bond geometry (Å, °)

**Table:**

*D*—H···*A*	*D*—H	H···*A*	*D*···*A*	*D*—H···*A*
N1—H1···O2	0.86 (1)	2.30 (1)	3.025 (2)	144 (2)
N2—H2···O2w^ii^	0.85 (1)	2.18 (1)	2.974 (2)	154 (2)
O1w—H11···O2^ii^	0.83 (1)	1.95 (1)	2.774 (2)	172 (2)
O1w—H12···O2w	0.83 (1)	1.98 (1)	2.799 (2)	169 (2)
O2w—H21···O1	0.83 (1)	1.86 (1)	2.694 (2)	177 (2)
O2w—H22···O1^iii^	0.83 (1)	1.97 (1)	2.771 (2)	163 (2)

Symmetry codes: (ii) −*x*+2, −*y*+1, −*z*+1; (iii) −*x*+2, −*y*, −*z*+1.

## References

[bb1] Barbour, L. J. (2001). *J. Supramol. Chem.* **1**, 189–191.

[bb2] Bruker (2009). *APEX2* and *SAINT* Bruker AXS Inc., Madison, Wisconsin, USA.

[bb3] Hunter, T. M., McNae, I. W., Liang, X., Bella, J., Parsons, S., Walkinshaw, M. D. & Sadler, P. J. (2005). *Proc. Natl Acad. Sci. USA*, **102**, 2288–2292. 10.1073/pnas.0407595102 PMC548988 15701702

[bb4] Lindoy, L. F., Mahinay, M. S., Skelton, B. W. & White, A. H. (2003). *J. Coord. Chem.* **56**, 1203–1213.

[bb5] Sheldrick, G. M. (1996). *SADABS* University of Göttingen, Germany.

[bb6] Sheldrick, G. M. (2008). *Acta Cryst.* A**64**, 112–122. 10.1107/S0108767307043930 18156677

[bb7] Westrip, S. P. (2010). *J. Appl. Cryst.* **43**, 920-925.

